# Extended Reality in Diagnostic Imaging—A Literature Review

**DOI:** 10.3390/tomography9030088

**Published:** 2023-05-24

**Authors:** Paulina Kukla, Karolina Maciejewska, Iga Strojna, Małgorzata Zapał, Grzegorz Zwierzchowski, Bartosz Bąk

**Affiliations:** 1 Department of Electroradiology, Poznan University of Medical Sciences, 61-866 Poznan, Poland; 2 Department of Adult Neurology, Medical University of Gdansk, 80-210 Gdansk, Poland; 3 Department of Medical Physics, Greater Poland Cancer Centre, 61-866 Poznan, Poland; 4 Department of Radiotherapy II, Greater Poland Cancer Centre, 61-866 Poznan, Poland

**Keywords:** extended reality, diagnostic imaging, virtual reality, radiology

## Abstract

The utilization of extended reality (ER) has been increasingly explored in the medical field over the past ten years. A comprehensive analysis of scientific publications was conducted to assess the applications of ER in the field of diagnostic imaging, including ultrasound, interventional radiology, and computed tomography. The study also evaluated the use of ER in patient positioning and medical education. Additionally, we explored the potential of ER as a replacement for anesthesia and sedation during examinations. The use of ER technologies in medical education has received increased attention in recent years. This technology allows for a more interactive and engaging educational experience, particularly in anatomy and patient positioning, although the question may be asked: is the technology and maintenance cost worth the investment? The results of the analyzed studies suggest that implementing augmented reality in clinical practice is a positive phenomenon that expands the diagnostic capabilities of imaging studies, education, and positioning. The results suggest that ER has significant potential to improve diagnostic imaging procedures’ accuracy and efficiency and enhance the patient experience through increased visualization and understanding of medical conditions. Despite these promising advancements, further research is needed to fully realize the potential of ER in the medical field and to address the challenges and limitations associated with its integration into clinical practice.

## 1. Introduction

Extended reality (ER) is a rapidly developing technology with the potential to revolutionize medicine. It allows for virtual and real-world environments to integrate, creating a new level of interaction (immersion) and user engagement [1]. In the field of diagnostic imaging (DI), it has the potential to streamline the diagnostic process, improve patient outcomes, and reduce healthcare costs [2]. ER is an umbrella term that encompasses various simulated reality technologies, including virtual reality (VR), augmented reality (AR), and mixed reality (MR). These technologies provide stereoscopic and three-dimensional (3D) immersion within an environment, as in VR, or are overlaid onto a real-world background, as in AR or MR [2,3].

**Figure 1:**
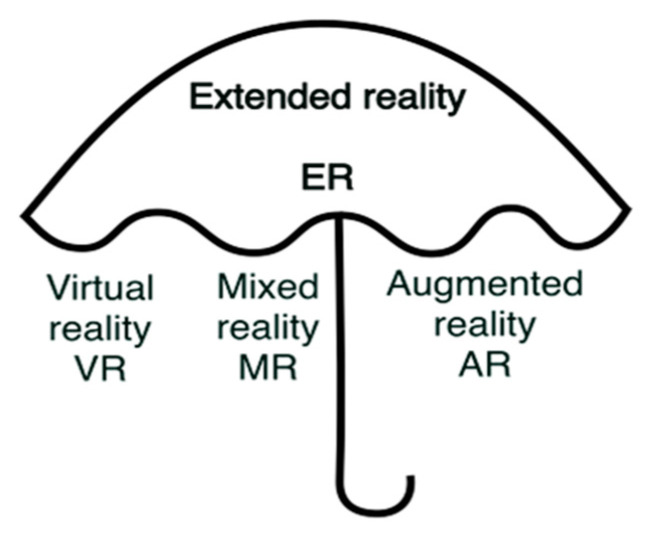
A graphical illustration of the classification of extended reality (ER) into subtypes of technologies such as: augmented reality (AR), mixed reality (MR), and virtual reality (VR). Self-developed illustration.

Extended reality (ER) is an example of rapidly developing technology with the potential to revolutionize various industries, including medicine. It is an umbrella term for simulating objects beyond the real world. It encompasses a range of technologies, including: (1) augmented reality (AR), (2) mixed reality (MR), and (3) virtual reality (VR). These technologies differ in their degree of immersion and interaction with the simulated environment. A graphic of the mix of these technologies is shown in Figure 1.

According to the analysis of the existing literature, there is a need for more standardization in the extended reality terminology, likely due to its ongoing and rapid advancements and potential uses. ER can be divided into three types: augmented reality (AR), mixed reality (MR), and virtual reality (VR). These technologies differ regarding the user’s ability to interact with the simulated environment and the degree of reality enhancement. The first type, augmented reality (1), is described as adding digital information to the real world, which the user can still see and interact with. Smartphone games, for example, commonly use this technology, such as in “Pokemon Go” (Niantic, Inc., San Francisco, CA, USA) or “The Witcher: Monster Slayer” (Spokko Inc., Warsaw, PL, USA). In medicine, AR can be used for guidance during procedures by superimposing both virtual and real images into the environment in real time [4,5]. The second type, mixed reality (2), combines digital and natural elements to create a new environment with which the user can interact in real time [6]. Virtual reality (3), the most well-known form of ER, creates an entirely immersive digital environment that replaces the real world [7]. It can be also used to provide a platform for remote training activities and scientific gatherings.

To create an extended reality environment, two main components are necessary. The first is a device that allows tracking of the user’s head and eye position, which provides information about the location of structures in the virtual environment. The second is the visual display of virtual elements from the user’s perspective. A smartphone, tablet, or and specialized glasses can still be effective for using AR technology, as they can provide a visual display of virtual elements from the user’s perspective. To experience VR and MR, a head-mounted device (HMD) or cave automatic virtual environment (CAVE) is required, allowing for complete immersion in the virtual environment by blocking out the real world and displaying visualizations [8,9]. This division can commonly be found in ER studies published after 2020 [10,11].

However, the implementation of ER technology in medicine is still in its early stages, and many challenges need to be addressed before it can be fully integrated with clinical practice. Diagnostic imaging is the cornerstone of diagnostics in modern medicine; using various techniques capable of “reading” the human body, helping in emergencies, and diagnosing diseases, its continuous development is important to improve the efficiency of diagnosing patients with cancer or cardiovascular diseases [11,12,13,14]. Extended reality is one of the fastest-growing technologies used in DI. In recent years, there has been a growing interest in these methods and an increasing number of attempts to implement ER elements in daily life and in other fields, such as medicine and education. The components of extended reality are virtual reality, augmented reality, and mixed reality, which create their own adequate environments [4,5,6,7,8]. This study aims to analyze scientific publications from the last ten years documenting the issues and applications of extended reality, including virtual reality (VR) and augmented reality (AR), in diagnosing and educating patients or medical students. The main objective was to present the possibilities of implementing ER in diagnostic imaging, including ultrasound [15,16,17,18,19,20,21,22], interventional radiology [23,24,25,26,27], computed tomography [28,29,30], positioning [31,32,33], and education [34,35,36,37,38,39,40,41], and also to present the role of the patient as a user during the examination or as a replacement in anesthesia and sedation procedures [42,43,44]. In summary, extended reality is a rapidly developing technology with a vast potential for use in various fields, including medicine, education, and entertainment. Further research is needed to fully explore and understand this technology’s potential and to create new and innovative ways of using it.

## 2. Materials and Methods

This review is based on the available literature from the PubMed and Scopus databases from the last 10 years (2013–2023). The searches included the following keywords and terms applied to titles and abstracts of full-length papers: [Extended reality] AND (virtual reality), [Journal] AND (mixed reality), [Journal] AND (augmented reality), and [Journal] OR “diagnostic imaging” [AllFields] OR “ultrasound” [All Fields]. We included articles and discussions that analyzed the role of augmented reality in diagnostic imaging. The search was limited to full-text articles, including electronic publications before printing. All the work collected was limited to human studies. The last PubMed search update for all sites was conducted on 24 January 2023. Papers in languages other than English and studies only available in abstract form were excluded from this analysis. Studies had to fulfill the following eligibility criteria to be selected for this review:

- Human research and English language only;
- Full articles and reviews;
- Using ER in diagnostic imaging.

**Figure 2:**
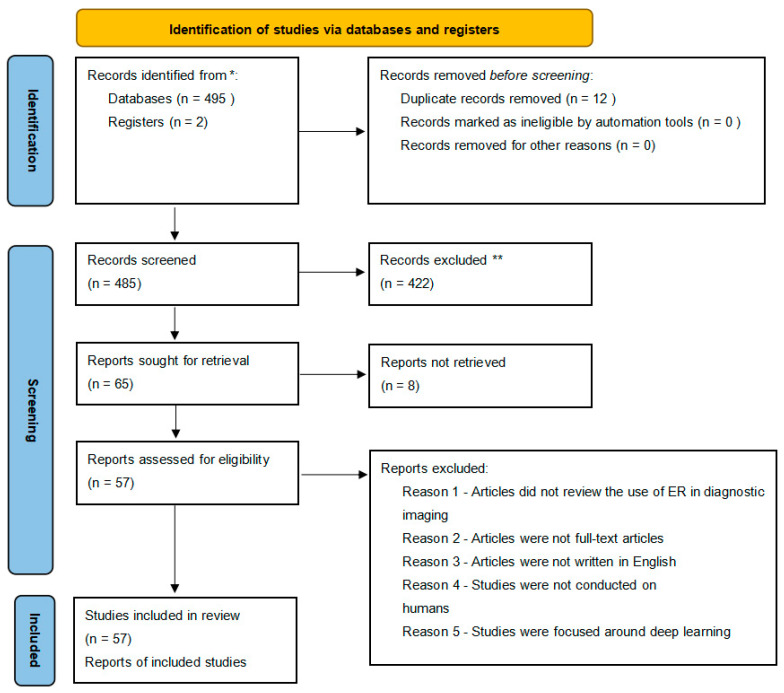
PRISMA analysis. * Records identified through PubMed and Scopus, ** Records excluded for not directly relevant.

According to a PRISMA [45,46,47] analysis, a total of 495 papers with eligible records were found in PubMed and Scopus, and 10 additional papers were extracted from their reference lists. After removing duplicates, 12,483 papers were screened for further analysis. We excluded 422 studies, as there were: 10 conference papers; 59 papers focused on surgery, radiotherapy, or neurology; 21 papers focused on virtual reconstruction; 5 papers focused on ER in other areas than diagnostic imaging, ultrasound, or education; and 5 papers about deep or machine learning. A detailed analysis is presented in Table 1. Finally, 57 studies could be included in the analysis for this review. All details are listed in the PRISMA workflow (Figure 2).

### 2.1. Extended Reality (ER) as a Replacement for Anesthesia and Sedation

Virtual reality (VR) has been proposed as a potential alternative to anesthesia and sedation in medical procedures such as burn treatment [42], interventional radiology [23], interventional oncology [43], or gynecology [44]. This approach, known as digital sedation, aims to use non-pharmacological and non-invasive VR tools to distract patients and reduce their perception of pain during medical procedures. Some studies have shown that patients perceive VR-based distraction methods as more engaging and immersive than other forms of distraction and report less pain during procedures. In their study, Yi-Ling Wang et al. [44] suggest that virtual sedation may also be applied in hysterosalpingography, a solution proposed as a protocol developed by the author. However, in some studies, no significant differences in pain intensity were found between patients using VR distraction and those who did not. Furthermore, more research is needed to fully understand the potential of VR as a replacement for anesthesia and sedation. Additionally, VR distraction may also be a solution to some of the current problems with anesthesia, such as high costs, emissions of nitrous oxide and other gases that can contribute to global warming, or contamination of operating rooms with these compounds [48].

### 2.2. Patient as a User during the Procedure

Recent studies have explored the use of virtual reality (VR) technology as an alternative to improve the patient experience during medical procedures. Nakarada-Kordic et al. [49] studied the differences between using a virtual simulation and a mock magnetic resonance imaging (MRI) simulation to reduce anxiety during the MRI procedure, including the level of comfort, the immersion of the patient in the simulation, and the patient’s mood during the simulation. VR in clinical practice can help reduce anxiety symptoms in patients, especially in imaging diagnostics. However, the study did not find statistically significant differences, but participants still found the VR simulation was very helpful in preparing for the examination. Another study by Vu et al. [50] suggests that computer-generated VR environments can safely and effectively provide simple, quick, quantitative assessments of movement dysfunction in patients being evaluated for Parkinson’s disease and can serve as an adjunct to brain imaging studies, such as [123I]FP-CIT SPECT/CT. Data on the patient’s movement limitations were collected and analyzed by simulating daily activities. VR devices such as controllers and head-mounted displays (HMDs) can provide accurate evaluations of parameters related to head and limb movements. Continuous data analysis using controllers can determine the position of the patient’s limbs and may be the basis for a new quantitative scale for evaluating patients with movement disorders.

In summary, recent research has shown that the use of virtual reality technology has the potential to improve the patient experience during medical procedures, especially in reducing anxiety during DI procedures and providing a quantitative assessment of movement dysfunction in patients with Parkinson’s disease. Studies have demonstrated that VR simulations can be helpful in preparing for examinations, and devices such as controllers and HMDs can provide accurate evaluations of patients’ movements. However, it should be noted that some studies did not find statistically significant differences between the use of VR and non-VR methods.

### 2.3. Positioning of the Patient in Medicine

Patient positioning for diagnostic examination is one of the essential and very crucial processes for obtaining a suitable image for further evaluation. VR technology using extended reality can be a valuable tool for proper patient positioning and preparation for diagnostics procedures [11,33,34,35,36,51].

The Oculus Rift system (Menlo Park, CA, USA) is a device for teaching positioning. In one of their studies, Sapkaroski et al. [48] showed that students who used VR in the process of learning positioning could better position the patient’s hand for radiographic examination compared to those who practiced using traditional methods. The advantage of learning positioning using VR is the ability to control bone alignment through the “layer tearing” option, which allows for examination of the positioned part of the body and alignment correction. Additionally, VR provided students with the ability to evaluate each step in the positioning process, which is considered a more effective and valuable form of education. Students’ survey responses were analyzed in another study comparing hand positioning learners using VR technology with the learning based on conventional patient positioning methods. One of the groups studied improved their skills using VR technology, while the other practiced traditional forms of learning. Students who did not use VR methods in learning required more time to improve their practical skills [12]. Many non-commercial and freely available virtual libraries, such as gVirtualXray (gVXR), (Bangor, UK) [52], are also used to develop positioning skills. Using these tools allows the user to see both correct and incorrect positioning, which minimizes the risk of exposure [33]. Overall, using VR technology in medical education can be a valuable tool for teaching positioning techniques and providing students with a more hands-on, interactive learning experience. It is worth noting that the use of VR technology in medical education is still in the early stages. Future research will be needed to confirm the benefits of this technology in the long term.

### 2.4. Education

For medical students and personnel, augmented reality methods such as VR and AR can provide immersive and interactive learning experiences to learn anatomy and patient positioning. With this approach, skills and knowledge in these areas can be effectively and efficiently improved without the need for actual patient interactions—that can ease the anxiety of a learner during the early stages of clinical education [38,40,41].

The DIVA system (Pasteur Institute and Institute Curie, Paris, FR), developed by the Mohamed El Beheiry uses VR and AR to visualize CT scans for medical education and training in diagnosing craniofacial trauma. The software allows the visualization of the scans in 3D and VR environments. According to a study by Bouaoud et al. [34], 92% of surveyed students were satisfied with using DIVA, and 83% noted its ease of use. The DIVA software also allows for detecting structures that would not be visible on a standard 2D image. Another system is the Magic Mirror (Guildford, UK), which is based on AR technology. It enables the learning of anatomy by superimposing 3D images of anatomical structures on the user’s body, creating the illusion of being able to see inside the body. A team developed the system at the University of Southern California. It consists of a head-mounted display worn by the user and a motion tracker to track the user’s movements. However, one of the main drawbacks of this system is its cost and availability in a commercial setting [35]. In a study undertaken by Weeks et al. [36], augmented reality technology was found to be particularly useful in learning anatomy, particularly the anatomy of the smaller body parts that can be difficult to learn due to their small size. Another example of an educational tool that uses AR technology is Second Life (SL) (San Francisco, CA, USA). SL is a virtual world that allows for e-learning and participation in meetings with users from different locations. Its unique features have made it an attractive tool for education and entertainment, while also raising concerns about addiction and questionable behavior. The platform is designed to simulate a fully realized virtual society, with users being able to interact with each other and participate in various events and activities. Virtual worlds can provide valuable and accessible educational tools, enhancing the learning experience in fields such as radiology [36,51]. Virtual reality simulators have been utilized for skill acquisition in robotic surgery. There have been attempts to enhance surgical robotic skills, and the preliminary outcomes are promising. Nevertheless, only one study involving actual patients has been conducted. Further investigation is necessary [39].

### 2.5. The Use of ER in Diagnostic Imaging

#### 2.5.1. Ultrasound (US)

The analyzed articles (n = 8) discussed using VR in gynecological, thoracic, and lung ultrasound applications. In ultrasound simulators in clinical settings, two different image generation methods are used: interpolation, in which 2D ultrasound images are generated based on patient data, and generative, in which computer models are generated manually [15]. Reijnders et al. [16] focused on developing a method for assessing the volume of uterine–placental vessels before conception and in the first trimester of pregnancy. The study involved 35 women. They used 3D Power Doppler ultrasounds in two VR systems—Barco I-Space and VR desktop. Petersma C. S. et al. [17] used 3D ultrasound datasets to detect fetal abnormalities in the first trimester of pregnancy. They identified women in the first trimester with a high risk for the fetus. In the study group, a traditional 2D ultrasound was performed in addition to a 3D VR ultrasound. The control group consisted of women who underwent a conventional examination.

M. Bazelmans et al. [18] highlighted the usefulness of the Barco I-space system in detecting additional renal arteries in the fetus. The system allows for a detailed evaluation of the course of additional arteries, their length and width, the number of branches, and their relationship with surrounding structures, which is limited with conventional ultrasound examinations. In another study, Pietersen et al. [19] presented the results of using a simulator for lung ultrasounds. Such studies have also been conducted to evaluate ultrasound simulators for transvaginal ultrasounds, including using the ScanTrainer (Intelligent Ultrasound, Cardiff, UK) as a training tool [20]. Jensen et al. [21] evaluated the FAST ultrasound simulator. The researchers pointed out the possibility of analyzing FAST results in simulated conditions while maintaining appropriate imaging standards.

There is growing interest in using virtual reality technology in diagnostic and ultrasound processes. As educational tools, VR methods can improve personnel confidence in performing the examinations. Additionally, ultrasound simulators can be valuable educational tools for students, residents, and sonographers [22,53,54].

#### 2.5.2. Computed Tomography

Using VR with diagnostic imaging techniques, such as computed tomography (CT), is becoming one of the main areas of interest nowadays. Mirhosseini et al. [28] proposed using VR as a tool for virtual colonoscopies (VCs) as a non-invasive and cost-effective screening procedure for colon cancer in which a fully integrated system with VR glasses allows for the capture of the pathology on a 3D image of the colon. The authors presented an immersive analytics system for VCs which enhances and improves the traditional desktop VC through the use of VR technology. Additionally, in another study, Kang et al. [11] used VR to analyze and evaluate heart abnormalities, specifically in the case of different types of defects; a double-outlet right ventricle converted the CT datasets of 12 heart specimens to stereoscopic images. The images were viewed and evaluated using MR goggles containing two lenses connected to a True 3D (Echopixel; Hewlett-Packard, Mountain View, CA, USA) using the mixed-reality system. The morphological features identified on the stereoscopic models were compared with findings at the macroscopic examination of the actual heart specimens. These studies’ results showed a high accuracy level; the average agreement coefficient between the pathological and imaging studies was 97%, as was the agreement between virtual and actual pathological samples. Furthermore, Sun et al. [30] demonstrated the usefulness of VR in minimizing errors in local lung segmentation. Studies conducted on data from eighteen sets of CT scans, in which errors occurred during the automatic segmentation, showed lower error rates than automatically generated segmentations (2.54 ± 0.75 mm before refinement vs. 1.11 ± 0.43 mm post-refinement, *p* < 0.001). The average user interaction time with the system was about 2 min.

#### 2.5.3. Interventional Radiology

In interventional radiology, diagnostic procedures (angiography, cholangiography, and phlebography) and therapeutic procedures (balloon angioplasty, thrombectomy, embolization, etc.) are performed. One advantage of interventional radiology is the ability to perform most procedures under local instead of general anesthesia, which shortens hospitalization times and reduces postoperative complications. However, a limitation of utilizing interventional radiology in medical procedures is the overexposure of the healthcare personnel to ionizing radiation, which is considerably higher than in other radiology departments. That is one reason why radiation protection specialists are increasingly seeking ways to reduce personnel exposure to ionizing radiation. Some authors believe virtual reality has the potential to visualize ionizing radiation doses in daily clinical practice through mixed reality and Monte Carlo simulations [51] or through virtual reality [27]. For example, a study by Yi Guo, Li Mao, et al. [55] estimated the dose delivered to a patient by designing a holographic visualization device which also displayed dose distributions in a room on HoloLens glasses or a monitor. A similar issue was investigated by Takeshi Takata, Susumu Nakabayashi, et al. [6].

Interventional radiology treatment rooms could be a particular application for VR, especially regarding virtual sedation, as discussed in a study by Cornelis, et al. [23]. The authors emphasized VR as an effective tool for reducing acute pain, anxiety, and discomfort. However, more research is needed to fully understand its neuropsychological basis and validate its effectiveness. Virtual sedation uses VR immersion to distract the patient from reality, creating a state of relaxation through enhanced sensory experiences.

Another practical application of VR was presented in a study by Matsuzaki S. et al. [24], who presented an example of virtual reality in interventional radiology to train medical staff in correctly using radiation shielding. The authors presented an application that allows the visualization of scattered radiation in an extended reality, which is difficult to visualize using traditional methods. A study of 33 healthcare workers showed that the application reduced the head exposure of the leading operator by 22–73%. A survey measuring satisfaction, confidence, attention, and accuracy showed high reliability of positive feedback. A study by Popovic B. et al. [25] focused on simulation training for coronary angiography and was conducted with 20 doctors. The results showed that the training participants had better results in radiation protection (collimation and reduced distance between the device and patient), shorter procedure times, and reduced contrast administration. There were no increased post-operative complications. These results suggest that using VR in simulation training is an effective way to improve skills. One of the key advantages of VR training is the ability to simulate real-life scenarios in a controlled environment. In a study by Jensen et al. [26], the researchers allowed trainees to practice the procedure in a safe and risk-free setting before performing it on real patients. The transfer effect of VR training to real-life catheterization labs has also been studied. Several studies have shown that trainees who underwent VR training performed better in real-life procedures compared to those who received traditional training methods. In addition, VR training has been shown to be particularly effective in improving trainees’ confidence levels and reducing their anxiety when performing procedures in the catheterization lab. Overall, VR training in coronary angiography has been shown to be an effective and valuable tool for medical professionals. It has the potential to improve patient outcomes, reduce medical errors, and increase the confidence and skills of trainees.

## 3. Discussion

In recent years, the potential of virtual and augmented reality technologies in medical education has received much attention [56]. They offer more interactive and engaging learning experiences, particularly in anatomy and patient positioning. The DIVA system is a successful tool for facial trauma diagnosis that uses VR to visualize data [34]. The Magic Mirror system uses AR to project 3D anatomy images onto the body for a more realistic understanding [35,57]. However, improvements in cost and accessibility are still needed [35]. AR technology has also proven helpful in teaching the complex anatomy of the head and neck [36]. In addition, as an e-learning tool, SL is used for virtual meetings and distance learning to enhance student engagement, in addition to VR and AR.

In procedures using ionizing radiation, one of the most valuable assets that ER provides is minimizing the doses used during procedures [24,25,55,56,57,58,59,60], with benefits to the patients and the staff. On the other hand, evaluating the cost-effectiveness of VR and AR in medical education is a crucial question when analyzing these technologies. While VR and AR provide a more interactive and engaging learning experience, the cost of the technology and maintenance must be considered. Based on the available literature, the effectiveness of using AR in ultrasound training, particularly in gynecology or breast applications, is a potential topic of discussion. Reijnders and others [16] discovered that the use of VR technology, specifically the Barco I-Space system, was effective in measuring the volume of maternal and fetal blood vessels before and during the first trimester of pregnancy. Similarly, incorporating 3D VR ultrasound data into traditional 2D ultrasound scans improved the ability to detect fetal abnormalities according to Petersma C. S et al. [17]. Although these VR and AR technology applications are focused on ultrasound, the potential exists to apply them to other medical fields as well. However, more research is needed to determine these technologies’ cost-effectiveness and overall effectiveness in the education and training of medical professionals. Another aspect worthy of discussion is the limitations and challenges in the implementation of VR and AR technology in ultrasound training. In addition, there is a call for the standardization of the VR and AR systems used for this purpose to facilitate comparison and to ensure the uniformity of the training. On the other hand, the potential of VR and AR to improve patient care through ultrasound imaging is also remarkable. For example, the DIVA system enables the visualization of CT scans in a 3D virtual environment, potentially helping healthcare providers better identify and diagnose injuries [34].

Augmented reality technologies such as AR, VR, and MR have gained attention in diagnostic imaging over the past years. Based on this review of 12 studies, researchers have shown interest in developing these technologies for DI purposes. However, there is a need for the standardization of concepts and knowledge about the benefits of AR, VR, and MR. Currently, there are varying definitions and understandings of what constitutes augmented reality.

In addition, when using VR technology in the medical field, the frequent occurrence of “cybersickness” is a significant concern. Symptoms of cybersickness, such as eye fatigue, nausea, and vomiting, can reduce the operator’s ability to concentrate and feel comfortable during procedures. Further research is needed to address these challenges [6,28]. Based on clinical studies, combining VR/MR technology with CT scanning also increases diagnostic potential. The risk of error and patient discomfort performing screening exams such as virtual colonoscopies could be significantly reduced by implementing these technologies in daily clinical practice. Virtual colonoscopy has many advantages over conventional colonoscopy; these include low cost, improved safety, shorter examination time, and appropriate measurement tools. Furthermore, the use of simulators of diagnostic devices, especially for MRI, in VR technology, may be beneficial for healthcare facilities that cannot install mockups of these devices to prepare patients for exams without exposing them to environmental risks. However, it should be noted that in all the aspects discussed in this review, the use of ER still needs data on the effectiveness of implementing virtual reality as a therapeutic or clinical modality, and there is an insufficient number of clinical studies using augmented reality.

The results of this analysis and their conclusions highlight the importance of integrating advanced medicine and technology. The use of virtual and augmented reality in diagnostic imaging can improve the accuracy and efficiency of examinations while simultaneously increasing patients’ comfort during these procedures. Further studies in this area will pave the way for creating innovative techniques and tools that can be applied in clinical practice, improving diagnostic processes and quality of care. This could increase productivity while reducing costs, which could be attractive to healthcare facilities.

## 4. Conclusions

The results of the modalities discussed indicate that integrating extended reality modalities such as AR, MR, and VR into clinical practice is beneficial. They expand the diagnostic capabilities of imaging examinations, education, and positioning. Currently, this research area has yet to be fully explored. Further research in this area will modernize diagnostic procedures and improve healthcare by creating new techniques and tools that can be used in clinical practice [58,59,60,61]. In conclusion, virtual and augmented reality technologies have the potential to transform medical education and patient care in diagnostic imaging.

## Figures and Tables

**Table 1:** Number of papers excluded from analysis.

Types of Research Excluded	N
Surgery	31
Virtual recon	21
Radiotherapy	12
Neurology	11
Conference paper	10
Forensics	7
Radiation oncology	5
Diagnostic without ER	5
Deep learning	4
Debate, abstract	3
Non-human	3

## Data Availability

The datasets analyzed in the current study are available from the corresponding author upon request.
